# Finding patterns in policy questions

**DOI:** 10.1038/s41598-022-21830-z

**Published:** 2022-11-22

**Authors:** Magda Osman, Nick Cosstick

**Affiliations:** The Centre for Science and Policy, Judge Business School, University of Cambridge, Cambridge, UK

**Keywords:** Psychology, Human behaviour

## Abstract

To help advance exchanges between science and policy, a useful first step is to examine the questions which policymakers pose to scientists. The style of a question indicates what the asker is motivated to know, and how they might use that knowledge. Therefore, the aggregate pattern of typical policy inquires can help scientists anticipate what types of information policy audiences desire. A dataset (n = 2972) of questions from policymakers collected over 10 years (2011–2021)—by the Centre for Science and Policy at the University of Cambridge—was classified into one of seven classes. In the main, the most popular questions posed by policymakers—within the public and private sectors—were those whose answers inform how to achieve specific outcomes—whether directly, or by providing a causal analysis which is instrumental to this process. Moreover, this seems to be a general aspect of policymakers’ inquiries, given that it is preserved regardless of the policy issue considered (e.g., Artificial Intelligence, Economy, or Health). Thus, maximizing the usefulness of the information that policymakers receive when engaging with scientists requires informing how to achieve specific outcomes—directly, or by providing a useful causal analysis.

## Introduction

Knowledge exchange activities between science and policy are driven by a need to address practical issues^1^. Several studies have highlighted barriers to effective translation from scientific evidence to policy^2,3^. In particular, scientists and policymakers often have different motivations and goals, which limit their collaboration^4^. Whilst the prospects for bringing these two communities’ motivations and goals into complete alignment are poor, scientists might reasonably gain a greater understanding of the motivations and goals of policymakers—and how the evidence they generate feeds into, or helps to achieve, them. Examining the questions which policymakers pose to scientists is instrumental to achieving this greater understanding. The ‘style’ of a question—the structure of the information sought (see below)—provides valuable indicators of what the asker is motivated to know, and what they might use that knowledge for^5,6^. Indeed, science-policy exchanges can often involve framing policy issues through a particular style of inquiry that articulates policymakers’ goals and the means of achieving them^7^. In light of this, the aim of this study is to examine the styles—and subject matters—of questions which policymakers pose to scientists, in order to expose any underlying patterns in these evidence requests. An understanding of these underlying patterns can potentially aid the integration of scientific evidence into policymaking through co-production^8^. Co-production requires a mutual understanding between scientists and policymakers^9^, which, in turn, requires clarity regarding any subject matter under discussion and the structure of the information sought. Furthermore, the integration of scientific evidence into policymaking is aided by tailoring evidence to the structure of the information sought by policymakers. To this end, the discussion will contain some advice for evidence tailoring.

Before considering the substantive lessons to be drawn from the literature on questions, a terminological confusion must be addressed. Within this domain, a universal agreement regarding the meanings of certain relevant terms has not yet been reached. This might cause confusion, as the same term can be deployed to refer to distinct features of questions and/or answers. For example, Pomerantz^5^ uses the term ‘content’ to refer solely to the subject matter that a question/answer concerns. By contrast—as noted by Pomerantz^5^—Graesser, McMahen, and Johnson^10^ use the term ‘content’ to refer both to a question’s subject matter and its style. When using antecedent studies to evidence the arguments in this paper, such terminological issues are disregarded in favour of the underlying point being made.

To begin, it is useful to consider two contrasting features of questions: subject matter and style. The subject matter of a sincere question indicates the information that the inquirer is interested in attaining, thereby indicating the kind of content which would be appropriate for a sincere answer^5^. For example, sincerely asking “what is net zero?” implies that one wants to know about the net-zero emissions goal. The style of a question—the structure of the information sought—indicates the understanding of the inquirer^11^, and what kind of answer is expected^5,12,13^. Asking a sincere question implies that the inquirer has enough of an understanding of the issue from which to build the question and interpret the answer, but does not know enough to make seeking the answer superfluous^11^. For instance, sincerely asking “what is net-zero?” requires that the inquirer has at least heard of the term ‘net-zero’ but does not have a complete understanding of its referent. Furthermore, the style of this question indicates that sincere answers should be structured as definitions. By contrast, sincerely asking “what do we need to do to achieve net-zero?” requires that the inquirer has a basic understanding of what net-zero is but not a complete understanding of how to bring it about. Moreover, the style of this question indicates that sincere answers should outline the procedure(s) which will bring about net-zero.

Earlier work in psychology^10,14,15^, linguistics^14^, and information science^5^ provided the foundation for the types of analyzes that have been used to develop taxonomies of questions. Importantly, question stems—such as “why…?”, “how…?”, “when…?”, and “what…?”—have not been the standard basis on which taxonomies are developed, because they are typically polysemous^12,17^. The ambiguity of question stems makes their application highly context-specific, hence why question classification systems have generally focused on question styles^12^.

The most practical approach to taxonomizing questions is to classify questions according to their style. Lehnert^13^ was the originator of this approach. Graesser, Person, and Huber^12^ later generated a simpler taxonomy of questions by style (the ‘Taxonomy of Question Styles’)—these questions were later grouped by the length of the expected answer by Graesser, McMahen, and Johnson^10^ (see Table 1). For example, “what does X mean?” was given as part of the abstract characterization (“abstract specification”) of the ‘definition’ style of question. Definition questions invite answers which specify the details—usually as long descriptions—that characterize a phenomenon or event. In contrast, “what caused some event to occur?” was given as the abstract characterization of the ‘causal antecedent’ style of question. Causal antecedent questions invite answers which outline the factors that brought about an event. Taxonomies of questions can be used to investigate applied scientific problems. In order to improve outcomes in a variety of domains, such taxonomies are used to understand how agents approach the task of structuring a problem or dilemma, what types of solutions they are expecting, and how their inquiries could be improved^6^.

**Table 1:** Two taxonomies of question styles.

The taxonomy of question styles			Taxonomy of policy questions		
Super ordinate category	Style category	Abstract specification	Super ordinate category	Style category	Abstract specification
Short answer	Verification	Did X occur?	Bounded answers	Verification/qualification	Is it the case that X is here? Did X event occur? Are Xs more inclined towards y? Is X a viable version of Y?
	Disjunctive	Is X or Y the case?		Comparison	What are the strengths and weaknesses of X? What are the costs and benefits of implementing X?
	Concept completion	Who? What? What is the referent of a noun argument slot?		Forecasting	Which areas would you foresee improving in the next 10 years? How likely is it that X will be popular in the future?
	Feature specification	What attributes does X have?			
	Quantification	How may are there of X?			
Long answer	Definition	What does X mean?	Unbounded answers	Example/explanation	Which X is more like Y? What would be a case where Y is like X? How does X work?
	Example	What is an example of X?			
	Comparison	How is X similar to Y?			
	Interpretation	What concept can be inferred from X?			
	Causal antecedent	What event caused X?		Casual analysis (antecedents or consequences)	What are the barriers that will prevent X from occurring? What are the effects of X if it is implemented now?
	Causal consequence	What are the consequences of X?			
	Goal orientation	What are the motives behind X’s actions?		Instrumental/procedural/enablement	How can we use X to make Y better? What would need to be incorporated to ensure that X is achieved? In what way can we measure X so that it can later be used to support y?
	Instrumental/procedural	What plan can allow for X to be achieved?			
	Enablement	What resource allows X to perform their action?			
	Expectational	Why did X event not occur?			
	Value judgement	What value does the responder place on X?		Explaining/asserting value judgments	How should the infrastructure available be used to produce x? How should X respond to y?
	Assertion	The inquirer makes a statement indicating lack of knowledge			
	Request/directive	The inquirer wants the responder to perform an action			

This table sets out the Taxonomy of Question Styles on the left, and the Taxonomy of Policy Questions on the right. The Taxonomy of Policy Questions was generated by applying the Taxonomy of Question Styles to the dataset, and moderating it—as explained in the methods section—in order to better capture the data.

The Taxonomy of Question Styles (see Table 1) has proved fairly popular. It has been successfully applied within the education sector^6,10,18,19^. It has also been used as a foundation of, and supplement for, arguments made by other education researchers^20–22^. Moreover, it has played an applied, foundational, and/or supplemental role in studies analyzing web search strategies^23–25^, consumer health-related inquiries^26^, interpersonal exchanges^27^, and interview settings^28^.

Through an analysis of the frequency of questions generated, this taxonomy has been used to determine what types of questions are most likely to appear in a particular domain. Such information feeds into proposals regarding what improvements are necessary to support an effective evidence exchange process. In the education domain—where the Taxonomy of Question Styles has been used most often—it has aided in identifying the types of inquires made by students, so that they can then be encouraged to formulate different styles of questions which enable a more substantive understanding of a topic^6^. For instance, often the efforts have been to shift students away from verification-style questions (“did X occur?”) to analytical questions—such as causal consequence-style questions (“why did X occur?”)—to develop deeper understanding.

The theoretical underpinning of work analyzing the quality of questions has largely been informed by the ‘Grasser-Person-Huber (GPH) Scheme’^6,12^. It proposes that there are three dimensions on which a question should be assessed. Firstly, style (“content”): the structure of the information sought. Secondly, question-generation mechanism: the psychological processes—goals, plans, and knowledge—which bring about a question. The GPH Scheme lists four question-generation mechanisms: reducing, or correcting, a knowledge deficit; monitoring common ground; social coordination of action; and control of conversation and attention. The scheme holds that these categories are orthogonal to the style categories, since—in theory—a style of question might be motivated by any question-generation mechanism. For example, an inquirer might ask “what are the consequences of academic freedom?” to address a deficit in their knowledge. Alternatively, the same question might be asked to monitor the extent to which they share common ground with the responder. The GPH Scheme’s final dimension of assessment is ‘degree of specification’: the extent to which the information sought is made clear. A highly specific question is clear regarding what information is sought. Whereas an under-specified question requires the responder to make inferences about which details are relevant to the inquirer.

Within cognitive psychology, associations have been made between the effective generation of questions and problem-solving ability, as well as the learning of complex material^6,29–32^. Within social psychology, improvement in ability regarding interpersonal exchanges has also been shown to be the result of asking good questions^33^, that can increase one’s likability^34,35^. Many of the efforts to improve cognitive functions (e.g. problem solving, critical thinking, memory, and text comprehension), by improving questioning, are based on two factors. Firstly, increasing the specificity of the question to ensure that the responder has the best chance of providing answers that are directly applicable. Secondly, encouraging ‘deep-reasoning questions’: those which direct the inquirer to ask questions that invite a causal analysis^6^. In essence, this involves considering the cause-effect relationships between variables to start examining the underlying structures that enable inferences to be made about what brings about observable outcomes^36,37^.

To date, there has been no empirical work examining the styles of questions that policymakers pose to scientific experts—including the types of questions that are asked, and the frequency by which they are asked. Once this is understood, it can be used to improve science-policy exchanges. Improvements can be made to the articulation of policy questions so that the value of the answers provided is maximized. Furthermore, scientists might find it easier to adapt their communication, in order to focus on the evidence that policy audiences want from them. To address this deficit, the present study analyzed policy questions that have been compiled by the Centre for Science and Policy (CSaP), at the University of Cambridge. CSaP is a knowledge brokerage which creates opportunities for public policymakers and academics—primarily scientists—to learn from each other. This is achieved through CSaP’s Policy Fellowships, as well as workshops, seminars, conferences, and professional development activities.

Applicants to CSaP’s main ‘Policy Fellowship Programme’ initially submit 3–6 questions which indicate the main policy problems they will explore throughout their Fellowship—along with a justification of their influence on public policy and their aims and objectives concerning the Fellowship. A panel of academics and civil servants review the applications and assess the candidates regarding how influential their role is, their intellectual capacity (to get the most out of 25 one-hour meetings with academics throughout the Fellowship), the extent to which their questions will interest academics, and the relevance of the contributions that might stem from addressing their questions. During an initial meeting with successful applicants, Fellows are provided with any feedback from the judging panel, including any suggested changes to their proposed questions. The Fellows are then asked to submit their finalized questions—if different from those submitted in their applications. Fellows spend five days in Cambridge for one-to-one meetings with academics. In general, Fellows visit Cambridge twice for this purpose, submitting 4–5 questions per trip (though sometimes the same questions are submitted for both trips). As a result of this process, CSaP has accumulated a database of policy questions submitted by over 400 Policy Fellows over 10 years.

The database was used to examine two properties of policy questions: (1) what frequent styles of questions are posed to expert scientists? (2) Is there a relationship between the subject matter and style of questions posed to expert scientists? By answering these questions, it is possible to build up a profile of what evidence policymakers invite scientific experts to provide, as well as what that evidence is applied to.

## Methods

At the time the analysis of the questions was conducted there were a total 4319 questions for the period of 05–09–2011 to 23–09–2021. These were generated from a total of 443 different Policy Fellowships taken up at CSAP. The questions were then cleaned. In particular, this involved removing statements and duplicate questions (posed by the same Policy Fellow over consecutive trips to Cambridge). Once this filtering had been applied, there were a total of 2927 questions from 409 Policy Fellows that were submitted for analysis. Each question contained the following details: (1) a unique number to identify it (1–2927), (2) whether the Policy Fellow was from a public or private sector organization (public, private), (3) the year that the question was submitted, and (4) the word length of the question.

To ensure an appropriate classification system was applied to the questions, the questions were classified using an iterative approach^38^. First, the Taxonomy of Question Styles was applied to all 2972 questions. This initial attempt to classify the policy questions served two purposes: to identify categories that are applicable to policy questions from the original taxonomy, and to identify new categories where needed. From this, a second taxonomy was developed, which included categories from the Taxonomy of Question Styles as well as some new ones.

The development of this revised taxonomy started from the principle that the question style categories deployed in a policy-specific taxonomy need to be useful to policymakers. This was inferred from each question style’s frequency in the coding of the questions. As shown in Table 2, those question styles with a low frequency were not carried over from the Taxonomy of Question Styles to the revised taxonomy (see Table 2). (Where possible, the revised taxonomy subsumed questions from these omitted categories into other style categories). In addition, the taxonomy included several other categories of question styles not present in the original taxonomy to reflect the kinds of questions that were frequently occurring—such as those that invited the inquirer to make forecasts.

**Table 2:** Frequency (%) of questions per style category (* indicates categories that were omitted in the development of the revised taxonomy).

Superordinate category	Subordinate category	Abstract specification	Frequency (%) of questions coded
Short answer	Verification	Did X occur?	172 (5.88)
	Disjunctive*	Is X or Y the case?	4 (0.14)
	Concept completion*	What?	5 (0.17)
	Feature specification*	What attributes does X have?	15 (0.51)
	Quantification	How may are there of X?	58 (1.98)
Long answer	Definition*	What does X mean?	23 (0.79)
	Example	What is an example of X?	102 (3.48)
	Comparison	How is X similar to Y?	58 (1.98)
	Interpretation*	What concept can be inferred from X?	21 (0.72)
	Causal antecedent	What event caused X?	267 (9.12)
	Causal consequence	What are the consequences of X?	234 (7.99)
	Goal orientation*	What are the motives behind X’s actions?	18 (0.61)
	Instrumental/procedural	What plan can allow for X to be achieved?	219 (7.48)
	Enablement	What resource allows X to perform their action?	545 (18.62)
	Expectational*	Why did X event not occur?	5 (0.17)
	Value judgement	What value does the responder place on X?	78 (2.66)
	Assertion*	The inquirer makes a statement indicating lack of knowledge	12 (0.41)
	Request/directive*	The inquirer wants the responder to perform an action	4 (0.14)

This table shows the frequency of the questions coded into each question style category when using the Taxonomy of Question Styles. It also shows the categories omitted from the Revised Taxonomy of Question Styles.

This ‘Revised Taxonomy of Question Styles’ (see Table 3) was then used to classify all 2927 questions, and two independent coders were used to validate the taxonomy. Each coded a subset of questions (n = 1224), the results of which are presented in Table 4. Applying a stringent process for agreement, with only exact matches recorded, both coders agreed on (n = 582) 47.55% of the questions. However, the coders identified the issue that the differences between some of the Revised Taxonomy of Question Styles’ categories are superficial. For example, instrumental/procedural and enablement have superficially different subjects and predicates, yet both drive at the same idea: they seek to identify things which can be used to achieve some goal. This violates the principle of ‘qualitative parsimony’^39^: categories should not be inflated beyond necessity. When taking this into account, along with the related point that some of the categories were broad enough to significantly span the specification of others, the next step was to determine how many of the questions coded revealed matches based on feasible overlaps. This identified an addition 331 matches between coders, which increased the total level of agreement to 74.59%. Categories with superficial differences were merged to achieve mutual exclusivity. The resulting ‘Taxonomy of Policy Questions’ (see Table 1) was then used to analyze the 2927 questions that are reported in the results section.

**Table 3:** The Revised Taxonomy of Question Styles.

Sub-ordinate category	Super ordinate category	Abstract specification
Verification/forced Choice (Y/N)	Short answer	Is it the case that X is here?
Quantification	Short answer	How many are there of X?
Qualifying Quantified Possibilities	Short answer	What reasons are there for needing to do X?
Value Judgments	Short answer	What value does the answerer place on an idea or advice? Which X is best?
Example	Long answer	What is an example or instance of the instance/event/behavior?
Comparison	Long answer	What are the costs and benefits (or strengths and weaknesses) of X?
Explanation (open & vague)	Long answer	What and how would X work?
Causal consequence	Long answer	What are the consequences of an event or state, given a set of other states or interventions?
Causal antecedent	Long answer	What state or event causally led/leads to an event or state or outcome of an intervention?
Forecasting	Long answer	What will happen by time scale X?
Explaining possibilities	Long answer	How is it that X could be used to achieve Y?
Explaining value judgments	Long answer	Why do you think X might be best if Y is used?
Instrumental/procedural/enablement	Long answer	What instrument/plan/strategy allows an agent to accomplish a goal? What methods of measurement are needed/could be used to detect X?

This table sets out the Revised Taxonomy of Question Styles, along with their abstract specifications.

**Table 4:** Frequency (%) of questions when classified according to the Revised Taxonomy of Question Styles.

Sub-ordinate category	Super ordinate category	Frequency of questions coded (n = 2927)	Subset of questions coded by coder 1 (n = 1224)	Subset of questions coded by coder 2 (n = 1224)
Verification/forced choice (Y/N)	Short answer	297 (10.15)	124 (10.13)	101 (8.25)
Quantification	Short answer	58 (1.98)	20 (1.63)	7 (0.57)
Qualifying Quantified Possibilities	Short answer	142 (4.85)	51 (4.17)	5 (0.41)
Value judgments	Short answer	98 (3.35)	38 (3.10)	185 (15.11)
Example	Long answer	164 (5.60)	69 (5.64)	28 (2.29)
Comparison	Long answer	99 (3.38)	32 (2.61)	34 (2.78)
Explanation (open & vague)	Long answer	274 (9.36)	129 (10.54)	176 (14.38)
Causal antecedent	Long answer	325 (11.10)	110 (8.99)	49 (4.00)
Causal consequence	Long answer	215 (7.35)	96 (7.84)	73 (5.96)
Forecasting	Long answer	158 (5.40)	63 (5.15)	76 (6.21)
Explaining possibilities	Long answer	628 (21.46)	304 (24.84)	181 (14.79)
Explaining value judgments	Long answer	161 (5.50)	64 (5.23)	19 (1.55)
Instrumental/procedural/enablement	Long answer	308 (10.52)	124 (10.13)	176 (14.38)

This table shows the frequency of the questions coded into each question style category—in its initial application and by each coder—when using the Revised Taxonomy of Question Styles.

The aim of the content analysis was to examine whether certain subjects lent themselves to particular question styles. This involved looking at the domains of the organizations to which the Policy Fellows belonged, to narrow down the subjects that informed the content analysis. There were seven common types of policy subject: Artificial Intelligence (AI), Economics and Finance, Education, Environment, Defense and Security, Health, and Technology/Manufacturing. From this, several associated topics were identified^38^. Each question was coded as “1” if a key subject—or associated terms for that subject—appeared at least once in the question. For some questions, multiple associated terms were found. To avoid skewing the data in such cases, the question was still coded as “1” to reflect that it was associated with a key subject (regardless of how many other associated terms were present in that question). Trends in subjects over time were not analyzed because the policy interests/positions of the Fellows are not controlled for. Consequently, some years the data is skewed towards different subjects by virtue of the interests/positions of the Fellows at the time.

## Results

A general point concerning the statistical analysis of the dataset was that—due to the nature of the dataset—the analyzes were ran to gather a general impression of the pattern of findings rather than to develop firm conclusions. Inferential statistics were used with caution, given that in many cases the data violated basic assumptions for running the test (e.g. independence).

To begin, while there is an uneven distribution of policymakers by whether they belonged to private (20%) or public sector (80%) organizations, a simple analysis indicated that there is no significant difference between the two groups by frequency of class of questions, *χ*^2^ (6, *N* = 2927) = 10.16, *p* = 0.12, Cramer’s V = 0.06. Given this, for the remainder of the analyzes performed, we collapsed across the two groups of policymakers. Generally, there were more questions that invited unbounded answers (76%) than bounded (24%), *χ*^*2*^ (1, *N* = 2927) = 890.96, *p* < 0.001. Running a further analysis indicated there were significant differences in the distribution of questions by the seven sub-ordinate categories, *χ*^*2*^ (6, *N* = 2927) = 110.54, *p* < 0.001, where the most frequently generated type of question was instrumental/procedural (see Table 5).

**Table 5:** Summary: frequencies (%) and mean word length (SD) of questions according to superordinate and subordinate categories of the Taxonomy of Policy Questions.

	Number of questions	Bounded answers			Unbounded answers			
		Verification/qualification	Comparison	Forecasting	Example/explanation	Casual analysis (antecedents or consequences)	Instrumental/Procedural	Explaining/asserting value judgments
Mean word length (SD)	2927	22.58 (15.67)	22.35 (15.71)	20.24 (8.22)	14.93 (9.70)	20.00 (9.39)	22.70 (12.88)	21.87 (11.24)
% Public	2342 (80.01)	343 (14.65)	79 (3.37)	132 (5.64)	348 (14.86)	455 (19.43)	734 (31.34)	251 (10.72)
% Private	585 (19.99)	97 (16.58)	20 (3.42)	27 (4.62)	90 (15.38)	84 (14.36)	201 (34.56)	66 (11.28)
% of total sample	2927	440 (15.03)	99 (3.38)	159 (5.43)	438 (14.96)	539 (18.42)	935 (31.95)	317 (10.83)

This table sets out the Taxonomy of Policy Questions. For each question-style, it shows: its mean word length (SD), the percentage of questions of this style which came from public policymakers, the percentage of questions of this style which came from private policymakers, and the percentage of questions of this style within the total sample.

Bounded (*M* = 22.53, *SD* = 15.66) questions are longer than unbounded questions (*M* = 20.42, *SD* = 11.49), *t* (2925) = 3.68, *p* < 0.001, *d* = 0.15, *BF* = 0.05, but the effect sizes and Bayes factor indicate that this is a weak difference. Looking at the average word counts for each of the seven classes of questions, example/explaining class of questions seems to be the outlier (*M* = 14.93, *SD* = 9.70). Applying the Bonferroni correction, when compared against the other unbounded answer types, the phrasing of example/explaining questions were significantly shorted than causal analysis questions, *t* (975) = 8.27, *p* < 0.005, *d* = 0.53, *BF*_13_ = 1.11, instrumental/procedural, *t* (1371) = 11.27, *p* < 0.005, *d* = 0.65, *BF*_25_ = 1.23, and explaining/asserting value judgments, *t* (753) = 9.08, *p* < 0.005, *d* = 0.67, *BF*_16_ = 2.41, verification/qualification, *t* (877) = 8.67, *p* < 0.005, *d* = 0.58, *BF*_15_ = 5.16, forecasting, *t* (595) = 6.15, *p* < 0.005, *d* = 0.57, *BF*_z_ = 1.94, comparison, *t* (535) = 6.04, *p* < 0.005, *d* = 0.67, *BF7* = 3.45. Overall, the word length of the question doesn’t appear to be indicative of the types of answers it invites.

There were two main ways in which the content of the questions was examined. The first was the frequency with which the seven key subjects appeared in the questions. The content analysis applied to identify the presence of any of the seven key subjects meant that approximately two thirds of the questions were coded by the seven main subjects (n = 1786/2927, 61%). Given that a question could contain multiple subjects, when taking this into account (n = 951/2972, 32.59%), the most commonly occurring subjects—occurring once in each question—were AI (n = 222/951, 23.34%) and Technology/Manufacturing (n = 205/951, 21.56%). A total of 595 questions had a combination of two subjects present, with the most common pairing being Environment and Economy/Finance (n = 180/595, 30.25%). A total of 193 questions had three subjects in combination, with the most common triple being AI, Environment, and Economy/Finance (n = 32/193, 16.58%). A total of 42 questions had four subjects in combination, with the most common quadruplet being AI, Technology/Manufacturing, Environment, and Economy/Finance (n = 7/42, 16.67%). A total of four questions contained five subjects and one question contained six subjects. None contained all seven subjects.

The next way in which the content of the questions was examined was how often the various question styles appeared within the set of questions for each subject. Since multiple subjects sometimes appeared within the same question, independence was violated, which prevented any categorical inferential analyzes. Nonetheless, it was possible to determine an overall impression of the most common class of questions which different subjects appeared in. All questions were classified (n = 1786) that were coded by subject into the seven different classes of questions, and this was then repeated for questions where the subject appeared only once in each question (n = 951)^38^. Classifying the questions by subject and by style on these two sets provided a basis for determining consistency in any patterns detected. Looking across both classification methods, the most common question-style for all seven main subjects, was instrumental/procedural questions (average 34%). This may not be a surprise given the base rate of this class of question. Where subjects differed was the second most common question class that they appeared in. When classifying all questions coded by subject, the second most common question class for six of the subjects was causal analysis (average 20%), with the exception of AI which was verification/qualification (16.67%). When classifying questions coded by subject appearing only once in a question, then the second most common class was causal analysis (average 21%) for AI, Environment, and Defence/Security. For Economics/Finance, Education, and Technology/Manufacturing the second most common class of question was example/explanation (average 19%), and for Health the second most common class of question was verification/qualification (23.14%). Overall, the indication from the examination of content by question class is that all seven subjects most commonly appeared in instrumental/procedural questions, thereafter the subjects appeared commonly in other unbounded questions-styles (e.g. causal analysis, example/explanation) with few appearing commonly in bounded question-styles (i.e. verification/qualification).

## Discussion

From a database of 2927 policy questions, that were classified according to a taxonomy that has its roots in research on the psychology of questions, we find that: (1) the two most frequent question-styles invite answers that address causal-analytic and instrumental/procedural matters; (2) regardless of the policy subject, the most common answer that policymakers invited informed instrumental/procedural questions. This indicates that the common questions that policymakers present in exchanges with scientists are deep-reasoning based questions, that aren’t just for reducing or correcting knowledge deficits, but for presenting knowledge for specific purposes—such as informing what policy interventions could be taken.

This has clear prescriptive implications for scientists who wish to participate in the co-production of policy—and specifically the integration of scientific evidence into policymaking. By tailoring their evidence to these most common policy question styles, scientists might reasonably hope to maximize their chances of success. The abstract specifications of these styles can be used for this purpose. For example, scientists might ask themselves: is there an obvious policy goal that this research might help to achieve? However, it might be necessary to update evidence tailoring to meet the specific interests of any policymakers they engage with.

The fact that the two most frequently generated classes of questions were causal-analytic (e.g. understanding mechanisms) and instrumental/procedural (e.g. interventions) reveals important information regarding the main motivations and interests of policymakers. Inviting answers that expose cause-effect relationships between variables is also key to examining the underlying structures that enable inferences to be made about what brings about observable outcomes^36,37^. This aligns closely with work in cognitive psychology that examines causal reasoning, which has shown consistent improvement in the way a causal-analytic representation can impact decision-making^37,40,41^, problem solving^42,43^, moral reasoning^44,45^, perception^46,47^, interpretation of statistical information^48,49^, and evidential reasoning^50,51^. Recently, the application of causal-analytic approaches has been extended to policymaking^52–56^. This work suggests that, in order to interpret the effects of a policy intervention, what is first needed is to decompose the context in which the intervention is introduced into its causal factors (i.e. the variables that will support as well as inhibit the efficacy of the intervention). Achieving this requires formulating questions that concern the mechanisms which can bring about change in a desired direction and what outcomes need to be measured to determine the causal link between the intervention and the outcome. Thus, there is a clear relationship between causal analysis and instrumental/procedural/enablement reasoning: the former is a means to the latter. Scientists wishing to engage with policymakers might keep this framework for interpreting the effects of a policy intervention in mind.

As the results show, policymakers do invite answers that are causal-analytic in nature, but they are half as popular as questions which invite answers that inform how to achieve specific outcomes (i.e. instrumental/procedural). This finding can be contextualized in light of the relationship between causal analysis and procedural reasoning: the most popular questions posed by policymakers—within the public and private sectors—were those whose answers inform how to achieve specific outcomes—whether directly, or by providing a causal analysis which is instrumental to this process. Given the importance of causal analysis in determining the potential success of policy interventions, scientists might consider framing their answers via causal-analytic terms. Furthermore, depending on the reception to their instrumental/procedural questions, policymakers may consider increasing the number of causal analysis-style questions they ask.

Policymakers’ preference for asking instrumental/procedural questions is also relevant to several of the academic literatures on policymaking. It is consistent with several characterizations of ‘evidence-based policy’. This concept has been characterized in a strictly means-end way and, more broadly, as the complex interaction of evidence and individual, professional, and political goals^57,58^. Both characterizations allow some role for policymakers’ preference for instrumental/procedural questions. The result also provides some backing for the claim that the scientific and policy communities are divided. In its weaker form, this simply amounts to the claim that scientists and policymakers have different goals and motivations^4^. In its stronger form, it amounts to the claim that scientists and policymakers constitute two distinct communities that are poorly connected, motivated by different incentives, operate under different rules, and suffer from communication problems^59^. In either form, this claim might explain the poor fit between researchers’ assembling and packaging of information and policymakers’ practical needs^60^. However, the stronger claim (the ‘two communities’ theory) has faced robust criticism. In particular, it is hard to provide a specific characterization of the theory’s titular communities which is consistent with the data^61,62^. Thus, perhaps this result might be more fruitfully associated with the weaker claim, as part of a nuanced account of science-policy interaction. Finally, as the previous paragraph indicates, this result could be of use in the policy studies project of developing ‘bridging instruments’: tools which help to bring academics and policymakers closer together^61^.

Other insights from the analysis of the questions suggest that more unbounded (long, open ended) questions were generated than bounded (short, closed) questions. Given that there were fewer classes of bounded question-styles to unbounded question-styles, one might think it inevitable that fewer bounded questions would be identified. However, previous work provides evidence which suggests that, independent of the number of categories of questions corresponding to short vs. long answer types—depending on the domain—more short answer types are generated than long answer types. Domains in which this seems to hold include: education^6^, interviewing^28^, and health inquiries^26^. Thus, the fact that more unbounded than bounded question-styles were generated may reflect the properties of the domain in which the questions were asked—the policy domain—rather than the taxonomic structure used to classify the questions. In the main, policymakers tended to ask questions directed towards detailed answers. Often this meant steering away from constraining answers to provide an estimate about a future outcome (forecasting), verifying a particular understanding of an issue (verification/qualification), or outlining the strengths/weaknesses costs/benefits of a particular issue (comparison). The lesson for scientists wishing to participate in the co-production of policy is that less constrained answers—regarding forecasting, verification/qualification, and/or comparisons—might be required to address policymakers’ needs.

A final point to consider concerns important barriers that limit potential science-policy exchanges, thereby retarding the co-production of policy. Since co-production requires a mutual understanding between scientists and policymakers^9^, understanding the needs and goals of one’s audience is an important barrier which must be surmounted. Splitting this task into understanding the subject matter under discussion and the structure of the information sought might aid its completion. Moreover, treating the process as iterative—whereby the answer sought from the question posed is the first step in a dialogue which establishes mutual grounds on which to then revisit the question and how it can be addressed—is also important. Another potential barrier is that scientists have concerns about the possible blurring of lines between acting in the capacity of providing expertise versus advocating^4,63,64^. The findings from this study suggest that this may be warranted, given that the most common type of answer which policymakers invited from scientists was one that involved suggestions for interventions (e.g. methods of measurement, plans of action, types of instruments) that serve particular goals. While addressing questions of this type may lead to more impact for the scientific knowledge provided, it may potentially draw scientists into making recommendations, rather than presenting policymakers with factors to consider.

## Supplementary Information

Supplementary Information 1.

Supplementary Information 2.

## Data Availability

The datasets generated and/or analyzed in the study are available in the Open Science Framework repository (https://osf.io/c978s/files/).
