# Supplementary material for: Finding patterns in policy questions
Source: Sci Rep. 2022 Nov 22;12:20126. doi: 10.1038/s41598-022-21830-z (PMC9684423; doi:10.1038/s41598-022-21830-z)
Supplement: Supplementary file 1 — Supplementary Information 1. [file 41598_2022_21830_MOESM1_ESM.docx]

**Supplementary file – Finding patterns in policy questions**

Magda Osman^1^, Nick Cosstick^1*
1^ The Centre for Science and Policy, Judge Business School, University of Cambridge

**Stage 1: Preparation of the questions for analysis**

From the period of 05-09-2011 to 23-09-2021 a total of 4319 questions were generated from a total of 443 different policy fellowships taking up by policy fellows at Centre of Science and Policy (CSaP), University of Cambridge, UK.

The first approach that was taken was to:

1. To identify the policy questions according to whether they were questions or instead statements.
2. Remove all repetitions of policy questions/policy statements. The policy fellowship scheme lasts 2 years, within which policy fellows have the opportunity to speak with academics across CSaP’s network. In many cases, policy fellows end up submitting the same policy questions/statements for each trip to meet with academics at Cambridge.
3. Determine the word count for each policy question/statement
4. Classify the policy fellows according to whether they belonged to private sector or public sector organisations, this classification was based on how the organisations themselves on websites of the organisation referred to the organisation.
5. Identify how many questions were submitted as subsidiary questions to the main policy questions and omit them so that only the main questions were included in the data set. For instance, many policy fellows provided 5-7 policy questions, but for each one there were also follow up qualification questions, or sub-components to the main question. The number of follow-up/sub-components per policy questions submitted were removed to make the data set manageable and to focus on the first main set of questions presented.

Also, of note, to protect the policy fellows’ anonymity, given that the questions often contained details that could identify the individual or their organization, the data set (OSF, https://osf.io/c978s/files/) we provide for public accessibility records the questions in abstract form. For example, if a question were “how is it possible to quantify the impact on social change in rural Spain, after introducing a new land tax?”, the question was recorded as “how is it possible to quantify the impact on X in Y, after introducing a new Z? Where possible the abstract representation of the questions was presented so that as many details remained without compromising the essential characteristics, but in many cases, the details had to be stripped back to ensure anonymity. However, to perform the initial screening of the questions, for the classification and content analysis the complete version of the questions was used.

Once the duplicates were removed (n = 941), this reduced the entire list from 4319 to 3378 policy questions and statements. Table 1 presents a summary of the break down frequency of individual policy questions and statements by the sector that the policy fellow is associated with at the time they took up the fellowship, and by those questions that were actually phrased as statements rather than questions.

Table 1. Summary findings from policy questions and statements by sector.

|  | Total policy submissions  (n = 3378) | Word length average | Just Questions  (n = 3092) | Word length  average | Statements  (n = 286) | Word length  average |
| --- | --- | --- | --- | --- | --- | --- |
| Public | 2535  (75.04%) | *M* = 20.85, *SD =* 12.96 | 2342  (75.74%) | *M* = 20.89, *SD =* 12.16 | 193  (67.49%) | *M* = 18.82, *SD =* 20.37 |
| Private | 630  (18.65%) | *M* = 19.93, *SD =* 13.49 | 585  (18.92%) | *M* = 20.42, *SD =* 13.44 | 45  (15.73%) | *M* = 11.12, *SD =* 9.59 |
| unknown | 213  (6.31%) | *M* = 16.52, *SD =* 10.61 | 165  (5.34%) | *M* = 19.26, *SD =* 9.64 | 48  (16.78%) | *M* = 3.71,  *SD =* 2.30 |

**Stage 2. Development of the Taxonomy of Policy Questions**

Each of the 2927 policy questions was coded using a linguistic taxonomy of questions. The starting point was Pomerantz (2005)^[[1]](#footnote-1)^ work which reviewed several taxonomies of question styles. The most valuable for analysing the policy questions in this data set was the ‘Taxonomy of Question Styles’ (see Table 2) (Graesser, McMahen, & Johnson, 1994)^[[2]](#footnote-2)^. The reason for choosing this taxonomy was that it classifies questions by the information/answers sought. This is appropriate for a context where policy fellows engage with academics based on transferal of knowledge/advice seeking, rather than a knowledge exchange in a dyadic interaction.

Having reviewed all 2927 policy questions, the next step was to submit the questions to the Taxonomy of Question Styles (Graesser et al, 1994). In initial process of classification, it was clear that some of the categories that were included in the Taxonomy of Question Styles were not frequently occurring enough to be included, and the examples of policy questions that were not captured by the taxonomy. This meant that new categories were formed, and other classes of questions were redefined to capture more precisely the styles of questions generated by the policy fellows.

Table 2. Frequency (%) of questions per style category (* indicates categories that were omitted in the development of the revised taxonomy)

| Superordinate Category | Subordinate Category | Abstract Specification | Frequency (%) of Questions Coded |
| --- | --- | --- | --- |
| Short Answer | Verification | Did X occur? | 172 (5.88) |
|  | Disjunctive* | Is X or Y the case? | 4 (0.14) |
|  | Concept Completion* | What? | 5 (0.17) |
|  | Feature Specification* | What attributes does X have? | 15 (0.51) |
|  | Quantification | How may are there of X? | 58 (1.98) |
| Long Answer | Definition* | What does X mean? | 23 (0.79) |
|  | Example | What is an example of X? | 102 (3.48) |
|  | Comparison | How is X similar to Y? | 58 (1.98) |
|  | Interpretation* | What concept can be inferred from X? | 21 (0.72) |
|  | Causal Antecedent | What event caused X? | 267 (9.12) |
|  | Causal Consequence | What are the consequences of X? | 234 (7.99) |
|  | Goal Orientation* | What are the motives behind X’s actions? | 18 (0.61) |
|  | Instrumental/Procedural | What plan can allow for X to be achieved? | 219 (7.48) |
|  | Enablement | What resource allows X to perform their action? | 545 (18.62) |
|  | Expectational* | Why did X event not occur? | 5 (0.17) |
|  | Value Judgement | What value does the responder place on X? | 78 (2.66) |
|  | Assertion* | The inquirer makes a statement indicating lack of knowledge | 12 (0.41) |
|  | Request/Directive* | The inquirer wants the responder to perform an action | 4 (0.14) |

In the initial first pass at applying Graesser at al’s (1994) adapted Taxonomy of Question Styles, only 1840 of the 2927 questions were classified, this meant that 1087 (37.13% of all questions) questions were left unclassified. Therefore, on this basis a careful screening was applied to then devise an adapted version of Graesser at al’s (1994) Taxonomy of Question Styles to capture the questions that were unclassified. The taxonomy was revised in two ways, some of the classes of questions were removed given the low numbers of questions in that category, and where possible the construction of the revised taxonomy attempted to subsume questions from the omitted class of questions into other categories. In addition, the taxonomy included several other class of questions not present in the original taxonomy to reflect the kinds of questions that were frequently occurring such as those that invited the inquirer to make forecasts.

Table 3 the Revised Taxonomy of Question Styles, adapted from Graesser et al. (1994). To help with the classification process, the abstract specification was amended to provide examples that could facilitate the classification process to suit the styles of questions generated by policy fellows. This taxonomy was applied to the set of 2927 questions, and the frequencies by different class of questions is presented in Table 4.

Table 3. The Revised Taxonomy of Question Styles

| Sub-Ordinate  Category | Super Ordinate  Category | Abstract Specification |
| --- | --- | --- |
| Verification/Forced Choice (Y/N) | Short Answer | Is it the case that X is here? |
| Quantification | Short Answer | How many are there of X? |
| Qualifying Quantified Possibilities | Short Answer | What reasons are there for needing to do X? |
| Value Judgments | Short Answer | What value does the answerer place on an idea or advice? Which X is best? |
| Example | Long Answer | What is an example or instance of the instance/event/behaviour? |
| Comparison | Long Answer | What are the costs and benefits (or strengths and weaknesses) of X? |
| Explanation  (open & vague) | Long Answer | What and how would X work? |
| Causal Consequence | Long Answer | What are the consequences of an event or state, given a set of other states or interventions? |
| Causal Antecedent | Long Answer | What state or event causally led/leads to an event or state or outcome of an intervention? |
| Forecasting | Long Answer | What will happen by time scale X? |
| Explaining Possibilities | Long Answer | How is it that X could be used to achieve Y? |
| Explaining Value Judgments | Long Answer | Why do you think X might be best if Y is used? |
| Instrumental  /Procedural  /Enablement | Long Answer | What instrument/plan/strategy allows an agent to accomplish a goal? What methods of measurement are needed/could be used to detect X? |

Table 4. Frequency (%) of questions when classified according to the Revised Taxonomy of Question Styles

| Sub-Ordinate  Category | Super Ordinate  Category | Frequency of Questions Coded  (n =2927) | Subset of Questions Coded by Coder 1  (n = 1224) | Subset of Questions Coded by Coder 2  (n = 1224) |
| --- | --- | --- | --- | --- |
| Verification/Forced Choice (Y/N) | Short Answer | 297 (10.15) | 124 (10.13) | 101 (8.25) |
| Quantification | Short Answer | 58 (1.98) | 20 (1.63) | 7 (0.57) |
| Qualifying Quantified Possibilities | Short Answer | 142 (4.85) | 51 (4.17) | 5 (0.41) |
| Value judgments | Short Answer | 98 (3.35) | 38 (3.10) | 185 (15.11) |
| Example | Long Answer | 164 (5.60) | 69 (5.64) | 28 (2.29) |
| Comparison | Long Answer | 99 (3.38) | 32 (2.61) | 34 (2.78) |
| Explanation  (open & vague) | Long Answer | 274 (9.36) | 129 (10.54) | 176 (14.38) |
| Causal Antecedent | Long Answer | 325 (11.10) | 110 (8.99) | 49 (4.00) |
| Causal Consequence | Long Answer | 215 (7.35) | 96 (7.84) | 73 (5.96) |
| Forecasting | Long Answer | 158 (5.40) | 63 (5.15) | 76 (6.21) |
| Explaining Possibilities | Long Answer | 628 (21.46) | 304 (24.84) | 181 (14.79) |
| Explaining Value Judgments | Long Answer | 161 (5.50) | 64 (5.23) | 19 (1.55) |
| Instrumental  /Procedural/Enablement | Long Answer | 308 (10.52) | 124 (10.13) | 176 (14.38) |

Two coders when then presented with a subset of the questions (n = 1224) approximately 40% of the total number of questions to independently classify. The frequencies of the 13 categories of questions by each coder are presented in Table 4, and the actual classification of the questions is also presented in full (see OSF file). Applying a stringent process for agreement with only exact matches recorded, both coders agreed on (n = 582) 47.55% of the questions.

When taking into account that some categories were not mutually exclusive (e.g. explanation and explaining possibilities, explaining possibilities and Instrumental/procedural/enablement), and that given the abstract specification for classes of questions were broad enough that they significantly spanned other classes of questions, the next step was to determine how many of the questions coded revealed matches based on feasible overlaps. This approach identified an addition 331 questions where there were matches between coders. Taking this into account, the total level of agreement increased to 74.59%.

This exercise also helped to determine how to improve the abstract specification of the questions, to ensure that the details were distinct enough to limit their ambiguity that misclassifications could occur. Moreover, it helped to identify ways in which to further refine this taxonomy to the most distinct classes of questions. (Quantification was not carried over due to its low frequency when coded by coder 1 and coder 2—in line with the method followed in the development of the Revised Taxonomy of Question Styles. Furthermore, the process of refining the categories into distinct classes was carried out with reference to the coded data, rather than *a priori*. For example, *a priori*, explanation and example may seem like conceptually separate categories, yet—in the coding—these were two categories which often generated errors—for one coder, a question was an example, and for the other it was an explanation). This led to the development of the Taxonomy of Policy Questions (see Table 6) which was used to reclassify all 2927 questions, and later used for all other analyses.

Table 5. Combinations of classes of questions were there is degrees of overlaps, and correspondingly the frequency of classifications by coder 1 and coder 2.

| Combinations of class of questions that were often misclassified (e.g. causal antecedent misclassified as causal consequent) | | Frequency of Classifications |
| --- | --- | --- |
| Explaining possibilities | Instrumental  /procedural/Enablement | 123 |
| Explaining possibilities | Explanation | 32 |
| Explaining possibilities | Causal Consequent | 16 |
| Explaining possibilities | Causal Antecedent | 15 |
| Explanation | Causal Antecedent | 21 |
| Explanation | Causal Consequent | 14 |
| Instrumental  /procedural/Enablement | Causal Antecedent | 24 |
| Instrumental  /procedural/Enablement | Causal Consequent | 11 |
| Example | Explanation | 34 |
| Qualifying quantified possibilities | Explaining possibilities | 11 |
| Value judgement | Explaining value judgments | 30 |

Table 6. Taxonomy of Policy Questions

| Super-Ordinate Category | Sub-  Ordinate Category | Abstract Specification | Example |
| --- | --- | --- | --- |
| Bounded Answers | Verification/  Qualification | Is it the case that X is here? Did X event occur? Are Xs more inclined towards y? Is X a viable version of Y? | Do groups generally make better decisions than individuals? What is the justifications for using groups to make decisions? |
|  | Comparison | What are the strengths and weaknesses of X? What are the costs and benefits of implementing X? | What are the costs and benefits of groups over individuals? |
|  | Forecasting | Which areas would you foresee improving in the next 10 years? How likely is it that X will be popular in the future? | How will group decision-making shape action in the next 10 years? |
| Unbounded Answers | Example/Explanation | Which X is more like Y? What would be a case where Y is like X? How does X work? | Can you illustrate situations where groups make better decisions? How does group decision-making work? |
|  | Casual Analysis (antecedents or consequences) | What are the barriers that will prevent X from occurring? What are the effects of X if it is implemented now? | What happens if groups end up making the wrong decisions? What brings about the need to use groups in situations of adversity? |
|  | Instrumental  /procedural/Enablement | How can we use X to make Y better? What would need to be incorporated to ensure that X is produced? In what way can we measure X so that it can later be used to support y? | What strategies can organisation X implement for group decision-making to occur? What are the methods and strategies by which individuals can be encouraged to work in groups? |
|  | Explaining/asserting Value judgments | How should the infrastructure available be used to produce x? How should X respond to y? | Why do you think groups are the best way to make decisions? |

**Stage 3: Basic analysis of the structure of the questions**

Having refined the process of developing an appropriate taxonomy at Stage 3 the Taxonomy of Policy Questions was used to classifying the 2927 questions. Table 7 presents the frequencies of the questions by question class, along with the means and standard deviations of the word lengths of the questions. In all subsequent presentations of the findings from the classification of the questions we collapsed across those questions generated by public sector and private sector policy professionals. This was because the analysis conducted to compare the generation of questions by class did not reveal significant difference between the two groups of policy professional (public sector, private sector, *χ^2^* (6, *N* = 2927) = 10.16, *p* = .12, Cramer’s V = .06.

Table 7. Summary frequencies (%), mean word length (SD) questions according to superordinate and subordinate categories of the Taxonomy of Policy Questions

|  |  | Bounded Answers | | | Unbounded Answers | | | |
| --- | --- | --- | --- | --- | --- | --- | --- | --- |
|  | Number of Questions | Verification/  Qualification | Comparison | Forecasting | Example/  Explanation | Casual Analysis (Antecedents or Consequences) | Instrumental  /Procedural | Explaining/  asserting Value Judgments |
| % of total sample | 2927 | 440 (15.03) | 99 (3.38) | 159 (5.43) | 438 (14.96) | 539 (18.42) | 935 (31.95) | 317 (10.83) |
| Mean word length (SD) | 2927 | 22.58 (15.67) | 22.35 (15.71) | 20.24 (8.22) | 14.93 (9.70) | 20.00 (9.39) | 22.70 (12.88) | 21.87 (11.24) |
| % Public | 2342  (80.01) | 343 (14.65) | 79 (3.37) | 132 (5.64) | 348 (14.86) | 455 (19.43) | 734 (31.34) | 251 (10.72) |
| % Private | 585  (19.99%) | 97 (16.58) | 20 (3.42) | 27 (4.62) | 90 (15.38) | 84 (14.36) | 201 (34.56) | 66 (11.28) |

In addition, it was possible the pattern of questions that were generated across the 10-year period, for each of the seven sub-ordinate categories of policy question styles. To do this the frequency with which each question style appeared in a year was recorded and then converted into a percentage of the total number of questions generated for that year. In this way, it was possible to determine the distribution of each question style by year. For all of the bounded question styles (Verification/Qualification, Comparison, Forecasting) along with Explaining/Asserting value judgments the distribution of the questions appeared to remain fairly stable across years (See Figure 1). The other three notable exceptions were the patterns for Instrumental/Procedural, Causal Analysis, and Example/Explanation style questions. The proportion of Instrumental/Procedural and Causal Analysis questions increased over years, whereas the proportion of Example/Explanation questions decrease over years.

Regression analysis. To examine the type of relationship between the volume of questions generated and time, polynomial regression analyses were performed on the three question styles: Instrumental/Procedural, Causal Analysis, and Example/Explanation (see Figure 1). We made the values entered into the regression analysis bounded and static because of the way values for each class were coded (i.e. as percentages). An initial test was performed to determine the presence of autocorrelation for the variables time and the generation of instrumental/procedural questions generated, the Durbin-Watson result (2.31) indicated a slight negative autocorrelation. Time predicted the proportion of Instrumental/Procedural by time period explaining 57% of the variance, and best fit by a linear regression model (β = .76, *t =* 3.38, *p* = .007), *F*(1, 10) = 12.11, *p* = .007, *R^2^ = .57, R^2^_Adjusted_ = .53*), with the closest next best fit being a power regression model (β = .60, *t* = 2.26, *p* = .05), and least fit by a quadratic regression model (β = .18, *t* = .17, *p* = .86). The same analysis was performed on the variable time and the proportion of Causal Analysis questions generated for each year. The Durbin-Watson result (1.72) indicated a positive autocorrelation. Time predicted the proportion of Instrumental/Procedural by time period explaining 62% of the variance, and best fit by a quadratic regression model (β = 2.32, *t =* 2.44, *p* = .04), *F*(1, 10) = 6.78, *p* = .019, *R^2^ = .62, R^2^_Adjusted_ = .54*), with the closest next best fit being a linear regression model (β = .59, *t* = 2.21, *p* = .06), and least fit by a power regression model (β = .35, *t* = 1.12, *p* = .29). When performing an initial analysis examining the relationship between time and example/explanation style questions generated for each year none of the regression analysis models significantly the data, linear (β = -.25, *t* = -.78, *p* = .47), quadratic (β = -.10, *t* = -.07, *p* = .95), power (β = -.08, *t* = -.22, *p* = .83). To be sure that only two of the seven class of questions reflect a relationship with time period, each of the remaining four questions were entered into a regression analysis, and none revealed any significant relationships captured by any of the polynomial regression models. Thus, from the analyses and as indicated in Figure 1, the two most commonly generated question styles overall, also significantly trended in the direction of increasing significantly over time, and were captured by different relationships (linear, quadratic). Though, it needs to be stated that the analyses were performed to determine a general impression of the relationship, and given the transformation of the data for it to be entered into the analysis, some caution is taken in drawing any firm conclusions from these patterns. Nonetheless, what this indicates, is that that out of the seven subordinate categories of questions, Causal analysis questions and Instrumental/Procedural questions are the most popular for both groups of policy professionals (Public, Private) and that they make substantially more of the total number of questions than any other styles generated over time.


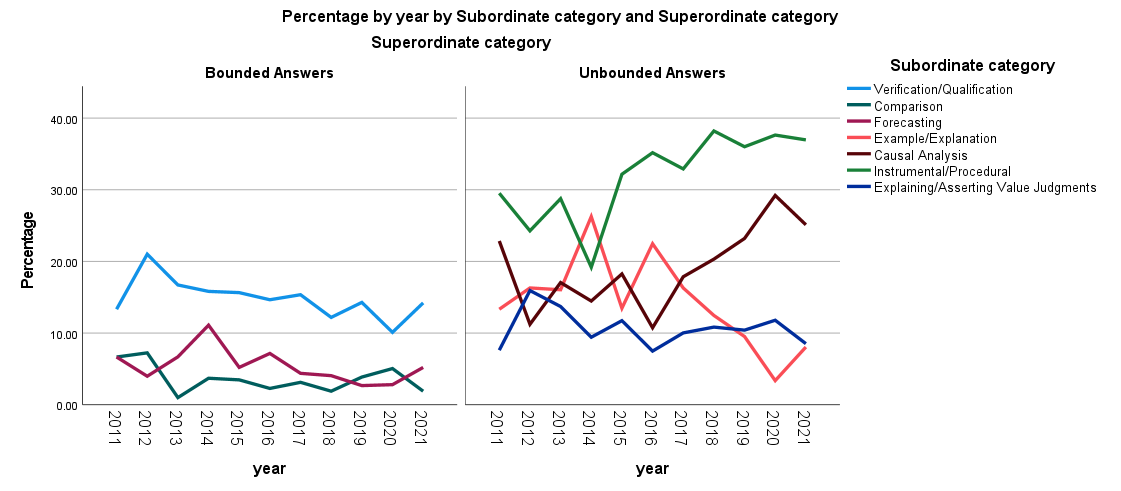


Figure 1. Percentage of questions by superordinate and subordinate categories of the Taxonomy of Policy Questions by time period

The next process was to look at the generation of question by the frequency of the most common question stems (How, When, Why, What, Could, Should, Can. To do this, the original file with the complete wording of the questions as they were presented by policy fellows were then coded based on the frequency by each question stem appeared in each question, according to how the question was classified. Table 8 present the findings of this method of coding the questions.

Table 8. Question stems by question class (raw numbers, %) (cells shaded from dark to light according to rank ordering 1^st^, 2^nd^ and 3^rd^ most common question stem).

|  | Verification/Quantification  (n =440) | Comparison  (n = 99) | Forecasting  (n = 159) | Example/Explanation  (n = 438) | Causal Analysis  (n = 539) | Instrumental/procedural  (n = 935) | Explaining/Asserting value  (n = 317) |
| --- | --- | --- | --- | --- | --- | --- | --- |
| HOW | 62 (14.09) | 17 (17.17) | 51 (32.08) | 201 (45.89) | 183 (33.95) | 607 (64.92) | 161 (50.79) |
| WHEN | 12 (2.73) | 4 (4.04) | 0 | 10 (2.28) | 12 (2.28) | 22 (2.35) | 14 (4.42) |
| WHY | 6 (1.36) | 1 (1.01) | 0 | 20 (4.57) | 3 (0.56) | 8 (0.86) | 0 |
| WHAT | 71 (16.14) | 85 (85.86) | 115 (72.22) | 231 (52.74) | 399 (74.03) | 348 (37.22) | 153 (48.26) |
| COULD | 41 (9.32) | 4 (4.04) | 7 (4.04) | 5 (1.14) | 13 (2.41) | 64 (6.84) | 12 (3.79) |
| SHOULD | 14 (3.18) | 1 (1.01) | 2 (1.26) | 1 (0.23) | 3 (0.56) | 13 (1.39) | 202 (63.72) |
| CAN | 101 (22.95) | 18 (18.18) | 25 (15.72) | 126 (28.77) | 143 (26.53) | 492 (52.62) | 66 (20.82) |

**Stage 4: Content analysis**

Not only were the policy questions classified using the Taxonomy of Policy Questions, to help examine patterns in the structure of the questions, but their content was also analysed. To do this, the initial approach taken was to look at the domains of the organizations that the policy fellows belonged to. This helped to narrow the general policy subjects that informed the content analysis, and make the process manageable. Moreover, by taking this approach we could feasibly justify the seven main subjects we could base our analysis on because they were directly connected to core domains that professionals were working in. Two coders where then asked to examine the questions while they conducted their initial classification of the questions, to record associated terms that appeared in the questions. This were then used to generate the associated terms that corresponded to a main subject, based on actual terms as they appeared in the policy questions. The details of the main subjects and associated terms are presented in Table 9.

Table 9. Main subjects and associated terms used to code the policy questions for content analysis

| Main Subjects | Associated terms | No. of associated terms |
| --- | --- | --- |
| Artificial Intelligence | “artificial intelligence” “digital” “smart” “ICT” “Big Data” “machine learning” “algorithms” “software” “cyber” “cryptocurrencies” “quantum computing” “data privacy” “blockchain” “internet of things” | 14 |
| Economics & Finance | “economics” “economy” “econometrics” “taxes” “finance” “financial” “incentives” “supply chains” “labour markets” “assets” “markets” | 11 |
| Education | “education” “educate” “schools” “schooling” “universities” “university” “teachers” “teach” “educators” “pupils” “students” “academia” “academics” “curriculum” | 14 |
| Environment | “environment” “climate change” “green energy” “sustainability” “sustainable” “decarbonisation” “pollution” “pollutants” “weather” “renewable energy” “carbon footprint” “anthropogenic” “fossil fuels” | 13 |
| Defense | “defence” “defense” “security” “threat” “combat” “deterrence” “decommission” “secure” “attack” “attacks” “terror” “terrorist” “terrorism” “extremism” | 14 |
| Health | “health” “medical” “medicine” “patients” “doctors” “medics” “diseases” “wellbeing” “mental health” “diagnosis” “obesity” “diet” “treatment” “diagnostics” “illness” “virus” “lifestyles” | 17 |
| Technology | “technology” “engineering” “engineer” “technological innovations” “industrial” “manufacturing” “construction” “infrastructure” “research and development” | 10 |

**Stage 5: Analysis of questions by content and structure**

The emergence of trends in subjects over time, an analysis of this kind was not conducted. There were two reasons for this. Firstly, the policy professionals were from the private as well as the private sector, which in itself would impact the types of subjects that would form the basis of the inquiries. Second, even if the content analysis was exclusively focused on public sector policy professionals, each year the policy fellows that take up a fellowship are not consistently representative of domains of public policy (e.g. Health, Defense, Justice, Education), and the variation in who takes up a fellowship in any one year means that for some years, it can be skewed towards one them (e.g. Education), and for another year skewed towards another (e.g. Health). So for these reasons, a trend analysis of subjects over years was not conducted.

There were seven common subjects: Artificial Intelligence (AI), Economics and Finance, Education, Environment, Defense and Security, Health, and Technology/Manufacturing. From this, several associated terms were identified (see Table 9). Using the subjects and associated terms identified the questions were then coded. Each question was coded as “1” if a key subject or associated terms for that subject appeared at least once in the question. For some questions multiple associated terms were found, so to avoid skewing the data, in any of these cases, the question was still coded as “1” to reflect that the question was associated with a key subject (regardless of how many other associated terms were present in that question). Table 10 presents the frequencies of the main subjects (or associated terms) appearing in each class of question. The table present the frequencies in two ways. First the frequencies by which subjects appeared in each class of questions where subjects could appear in combination in a question (e.g. AI and Technology/Manufacturing both appearing in a question classed as ‘Comparison’). Second, the frequencies by which a subject appeared in a question, excluding all questions were combination of subjects appeared in a single question.

Table 10. Frequency by which each of the main subjects appeared in each question class, shaded cells from darkest to lightest indicate the 1^st^, 2^nd^ and 3^rd^ most common classes of questions where each subject appeared

|  |  | Bounded Answers | | | Unbounded Answers | | | |
| --- | --- | --- | --- | --- | --- | --- | --- | --- |
|  | Number of questions | Verification/  Qualification | Comparison | Forecasting | Example/  Explanation | Casual Analysis (antecedents or consequences) | Instrumental  /procedural | Explaining/  asserting Value judgments |
| Classification of all questions coded by the 7 key subjects regardless of how often they appeared alone or in combination in each question (%) | | | | | | | | |
| AI | 486 | 81 (16.67) | 9 (1.85) | 32 (6.58) | 75 (15.43) | 58 (11.93) | 175 (36.01) | 56 (11.52) |
| Economics & Finance | 508 | 81 (15.94) | 13 (2.56) | 23 (4.53) | 62 (12.20) | 127 (25.00) | 148 (29.13) | 54 (10.63) |
| Education | 201 | 30 (14.93) | 6 (2.99) | 15 (7.46) | 31 (15.42) | 35 (17.41) | 62 (30.85) | 22 (10.95) |
| Environment | 610 | 105 (17.21) | 21 (3.44) | 29 (4.75) | 59 (9.67) | 122 (20.00) | 199 (32.62) | 75 (12.30) |
| Defense & Security | 311 | 39 (12.54) | 10 (3.22) | 8 (2.57) | 29 (9.32) | 62 (19.94) | 125 (40.19) | 38 (12.22) |
| Health | 289 | 41 (14.19) | 9 (3.11) | 18 (6.23) | 38 (13.15) | 48 (16.61) | 101 (34.95) | 34 (11.76) |
| Technology & Manufacturing | 506 | 60 (11.86) | 11 (2.17) | 47 (9.29) | 56 (11.07) | 81 (18.58) | 158 (34.78) | 52 (12.25) |
| Classification of questions coded by the 7 key subjects where each subject only appears once in a question (%) | | | | | | | | |
| AI | 205 | 30 (14.63) | 7 (3.41) | 17 (8.29) | 28 (13.66) | 40 (19.51) | 63 (30.73) | 16 (9.76) |
| Economics & Finance | 86 | 11 (12.79) | 3 (3.49) | 10 (11.63) | 16 (18.60) | 11 (12.79) | 26 (30.23) | 9 (10.47) |
| Education | 118 | 16 (13.56) | 4 (3.39) | 7 (5.93) | 21 (17.80) | 19 (16.10) | 36 (30.51) | 15 (12.71) |
| Environment | 98 | 9 (9.18) | 2 (2.04) | 2 (2.04) | 12 (12.24) | 22 (22.45) | 33 (44.90) | 7 (7.14) |
| Defense & Security | 101 | 15 (14.85) | 1 (0.99) | 4 (3.96) | 10 (9.90) | 22 (21.78) | 34 (33.66) | 15 (14.85) |
| Health | 121 | 28 (23.14) | 4 (3.31) | 8 (6.61) | 7 (5.79) | 15 (12.40) | 43 (35.54) | 16 (13.22) |
| Technology & Manufacturing | 222 | 45 (20.27) | 4 (1.80) | 16 (7.21) | 46 (20.72) | 23 (10.36) | 72 (32.43) | 16 (7.21) |

By coding the questions in two ways, namely according to whether a subject appears exclusively in a question, or in combination with others, it is possible to determine how consistent any patterns regarding where the subjects most commonly appear. For instance, it could have been the case that Defense and Security questions are most commonly structured as bounded questions than unbounded questions. In fact, from the patterns revealed in Table 10, all seven subjects were most commonly appearing in the Instrumental/Procedural class of questions. This may not be a surprise given that this class of questions is also the most commonly generated of all. Also, the proportion of times that the subject appeared in Instrumental/Procedural class of questions relative to the others, was about a third for all seven subjects; subjects appearing alone and in combination (*M =* 34.08%, *SD* = 3.64), subjects appearing only once per question (*M =* 34.44%, *SD* = 5.15). The same cannot be said for the second most common class of question that subjects appeared in, though the proportion of times a subject appeared in the next most common class of question, regardless of what class of question, was approximately a fifth; subjects appearing alone and in combination (*M =* 19.17%, *SD* = 2.93), subjects appearing only once per question (*M =* 20.57%, *SD* = 2.01). Where subjects could appear anywhere and in combination, the second most common class of question they appeared in was Causal analysis class of questions, with exception of AI which appeared in Verification/Quantification class of questions. When subjects appeared in a question only once (not in combination), then the pattern is mixed (see Table 10). When it came to the third most common question class that subjects appeared in, it was even harder to ascertain any consistent patterns across the full and reduced sets, other than they were more often than not bounded questions.

Overall, there are three broad conclusions that can be drawn from the pattern of findings. The Instrumental/procedural style is where subjects most commonly appeared. While not systematic regarding the second most common style of question subjects appeared in, the second most common question styles were predominately ones that invited unbounded answers, and the third most common invited bounded answers.

The next step was to examine the subjects by the frequency of question stem, this was another way to also look at possible patterns where subjects might be more commonly associated with particular features of the structure of questions. Table 11 presents the frequencies by which the main seven question stems appear in each of the seven main subjects, where the frequencies are separated out by question where the subjects appeared alone or in combination, and where they appeared exclusively per question. Overall, regardless of whether the full set, or reduced set of questions are examined, the three most common question stems are How, What and Can. The ordering of these questions by rank popularity (i.e. 1^st^, 2^nd^, 3^rd^) does vary by subject. When looking at the full set, then for AI, Economics & Finance, Education, Environment, and Technology & Manufacturing, the ordering is What (*M =* 48.78%, *SD* = 2.38), How (*M =* 42.45%, *SD* = 0.98) and Can (*M =* 32.53%, *SD* = 1.20). When looking at the reduced set, then for AI, Economics & Finance, Education, Environment, and Technology & Manufacturing, the ordering is What (*M =* 46.28%, *SD* = 2.87), How (*M =* 40.11%, *SD* = 4.53) and Can (*M =* 35.43%, *SD* = 3.84). It is only the subjects Defense & Security and Health where the frequency of patterns differs, in both the full and reduced set.

Table 11. Frequency of question stems by each of the 7 subjects (cells shaded from dark to light according to rank ordering 1^st^, 2^nd^ and 3^rd^ most common question stem).

| Question stems by 7 subjects regardless of how often they appeared alone or in combination in each question (%) | | | | | | | |
| --- | --- | --- | --- | --- | --- | --- | --- |
|  | AI  (n =486) | Economics/Finance  (n = 508) | Education  (n = 201) | Environment  (n = 610) | Defense & Security  (n = 311) | Health  (n = 289) | Technology/  Manufacturing  (n = 506) |
| HOW | 205 (42.18) | 210 (41.34) | 89 (44.27) | 256 (41.97) | 161 (51.17) | 134 (46.37) | 215 (42.49) |
| WHEN | 20 (4.12) | 6 (1.18) | 3 (1.49) | 14 (2.29) | 4 (1.28) | 4 (1.38) | 7 (1.38) |
| WHY | 7 (46.71) | 5 (0.98) | 2 (1.00) | 3 (0.49) | 3 (0.96) | 6 (2.08) | 4 (0.79) |
| WHAT | 227 (46.71) | 255 (50.20) | 92 (45.77) | 289 (48.85) | 133 (42.77) | 127 (43.94) | 265 (52.37) |
| COULD | 26 (5.49) | 36 (7.87) | 13 (6.48) | 45 (7.38) | 21 (6.75) | 15 (5.19) | 30 (5.93) |
| SHOULD | 37 (7.61) | 39 (7.68) | 17 (8.46) | 46 (7.54) | 35 (11.25) | 33 (11.42) | 7 (9.29) |
| CAN | 161 (33.13) | 157 (30.91) | 67 (33.33) | 191 (31.31) | 166 (37.30) | 95 (32.87) | 172 (33.99) |
| Question stems by the 7 subjects where each subject only appears once in a question (%) | | | | | | | |
|  | AI  (n =222) | Economics/Finance  (n = 101) | Education  (n = 86) | Environment  (n = 121) | Defense & Security  (n = 98) | Health  (n = 118) | Technology/Manufacturing  (n = 205) |
| HOW | 92 (41.42) | 41 (40.59) | 38 (44.18) | 52 (42.98) | 59 (60.20) | 57 (48.31) | 85 (41.46) |
| WHEN | 11 (4.95) | 1 (0.99) | 0 | 5 (4.13) | 1 (1.02) | 2 (1.69) | 2 (0.98) |
| WHY | 4 (1.80) | 3 (2.97) | 0 | 2 (1.65) | 1 (1.02) | 4 (3.39) | 1 (0.49) |
| WHAT | 94 (42.34) | 50 (49.50) | 40 (46.51) | 53 (43.80) | 41 (41.84) | 47 (39.83) | 101 (49.27) |
| COULD | 6 (2.70) | 9 (8.91) | 6 (6.98) | 13 (10.74) | 6 (6.12) | 6 (5.08) | 13 (6.34) |
| SHOULD | 10 (4.50) | 14 (13.86) | 8 (9.30) | 9 (7.44) | 8 (8.16) | 15 (12.71) | 15 (7.32) |
| CAN | 84 (37.84) | 31 (30.69) | 28 (32.56) | 38 (31.40) | 42 (42.86) | 40 (33.89) | 71 (34.63) |

1. Pomerantz, J. (2005). A linguistic analysis of question taxonomies. *Journal of the American Society for Information Science and Technology*, *56*(7), 715-728. [↑](#footnote-ref-1)
2. Graesser, A.C., McMahen, C.L., & Johnson, B.K. (1994). Question asking and answering. In M.A. Gernsbacher (Ed.), Handbook of psycholinguistics (pp. 517–538). San Diego: Academic Press. [↑](#footnote-ref-2)
